# Supplementary material for: Extracellular vesicles improve GABAergic transmission in Huntington's disease iPSC-derived neurons
Source: Theranostics. 2023 Jun 26;13(11):3707–24. doi: 10.7150/thno.81981 (PMC10334823; doi:10.7150/thno.81981)
Supplement: Supplementary file 1 — Supplementary figures and tables. [file thnov13p3707s1.zip › supp data/Supplementary data.docx]

**Supporting Information for**

**Extracellular vesicles improve GABAergic transmission in Huntington’s disease iPSC-derived neurons**

**Authors:**

Margarida Beatriz^1,2^, Ricardo J. Rodrigues^1,2^, Rita Vilaça^1,2^, Conceição Egas^1,2,3^, Paulo S. Pinheiro^1,4^, George Q. Daley^5,6^, Thorsten M. Schlaeger^5,6^, Nuno Raimundo^7^, A. Cristina Rego^1,8^*, Carla Lopes^1,2,7^*

Supplementary Data


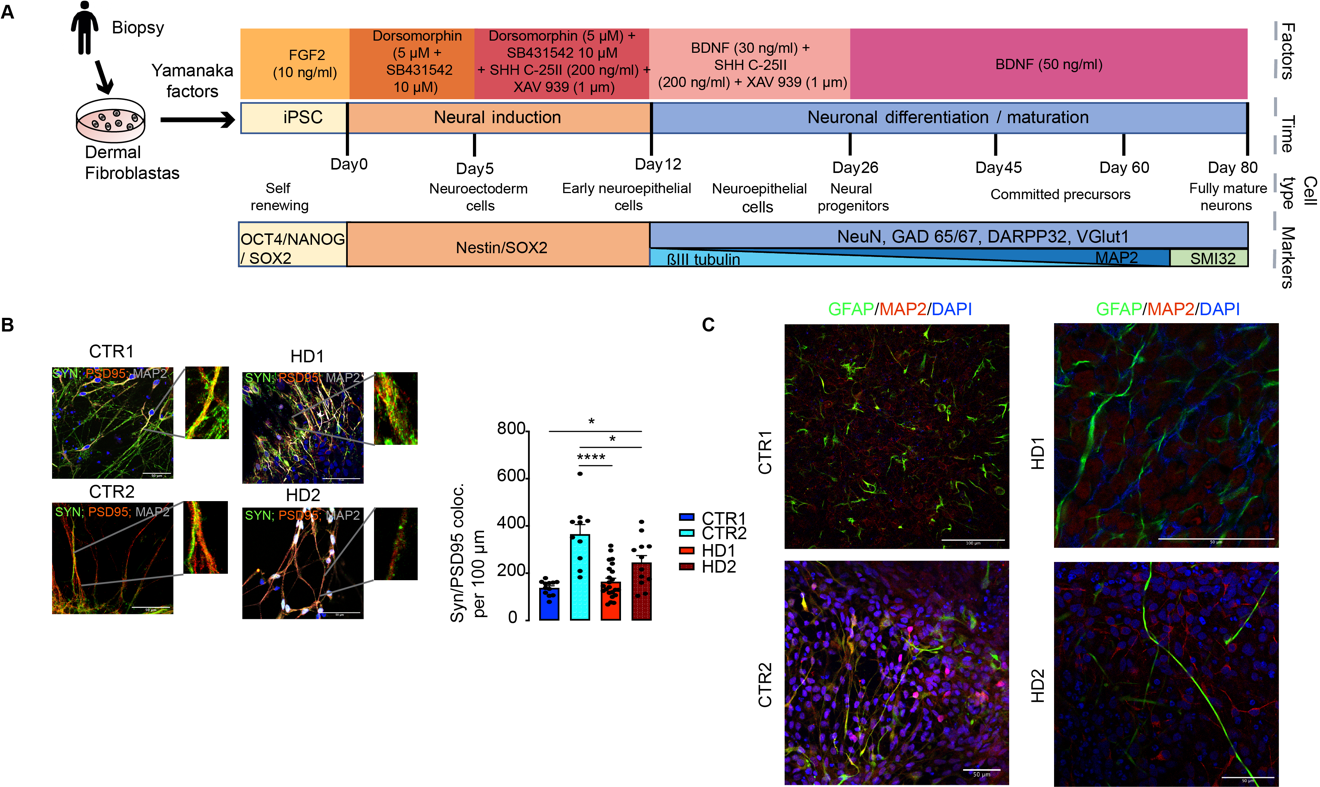


**Figure S1.** Differentiation protocol for striatal-like neurons.

(A) The iPSC markers include OCT4 and NANOG, and SOX2 is still present as neuroectoderm cells start to appear during neuronal induction, together with Nestin. Neuronal differentiation gives origin to neuroepithelial cells and βIII-tubulin is present in immature neurofilaments, whereas MAP2 is present as long as neurofilaments mature. Cultures of neuronal progenitors with a striatal lineage present NeuN, GAD 65/67, DARPP32, and VGlut1 as markers. SMI32 is present in fully mature neurons at day 80 of differentiation. (B) Mature striatal-like neurons (MAP2^+^) presented presynaptic (synaptophysin) and excitatory postsynaptic (PSD95) markers; colocalization of synaptophysin with PS95 in mature striatal-like neurons. (C) Glia marker (GFAP) is present in neuronal (MAP2+) cultures at day 80. Bar plots represent mean ± S.E.M. Statistical analysis: one-way ANOVA followed by Bonferroni multiple comparisons test: * p < 0.05, **** p < 0.0001.


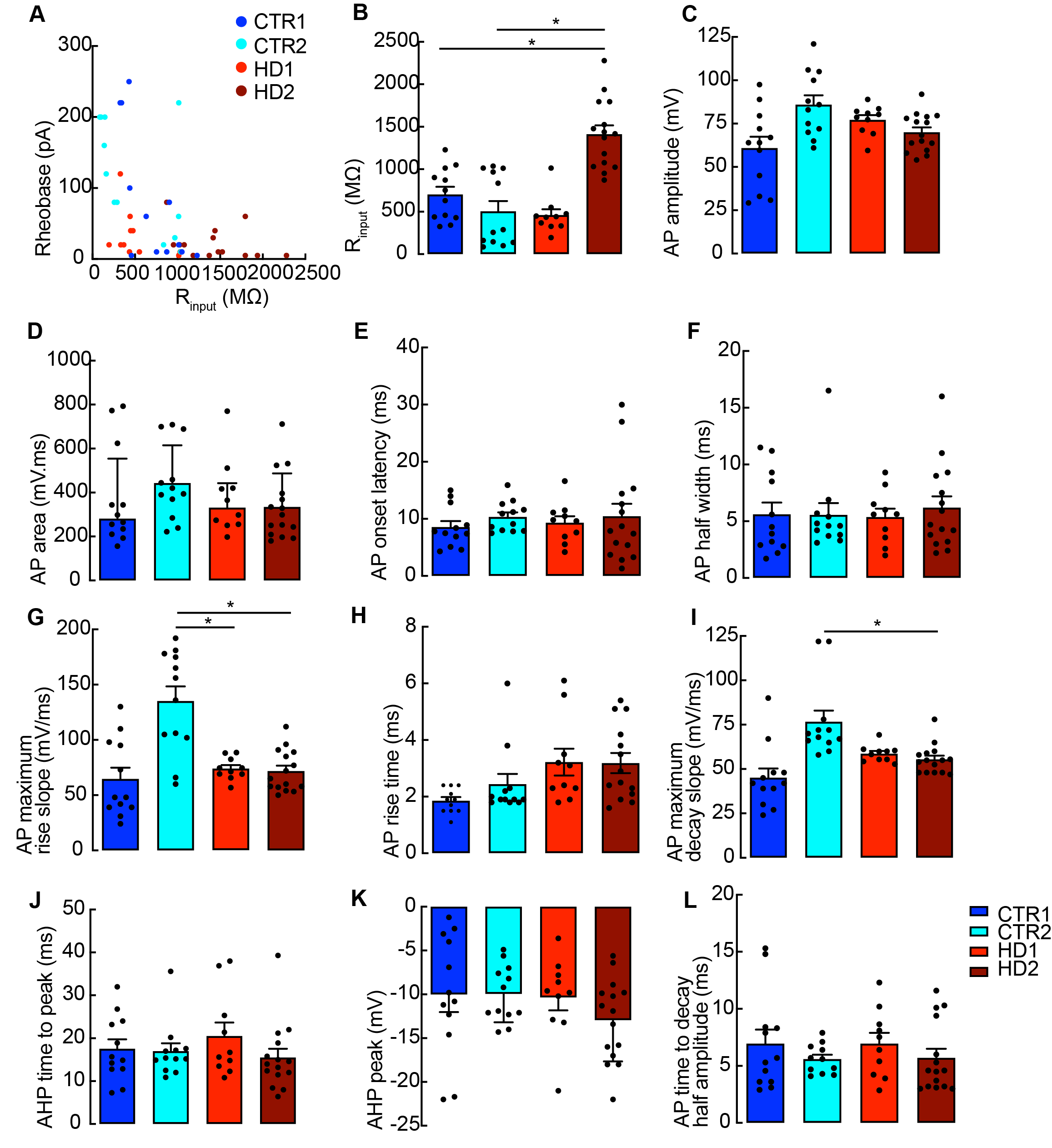


**Figure S2.** Electrophysiological properties of HD-derived neurons and CTRs.

Bar plots showing (A) Rheobase , (B) Input Resistance, (C) action potential amplitude, (D) AP area, (E) AP onset latency, (F) AP half-width, (G) AP maximum rise slope, (H) AP rise time, (I) AP maximum decay slope, (J) after-hyperpolarization potential (AHP) time to peak, (K) AHP peak and (L) AP time to decay half amplitude. One-way ANOVA followed by Tukey's multiple comparisons test: * p < 0.05.


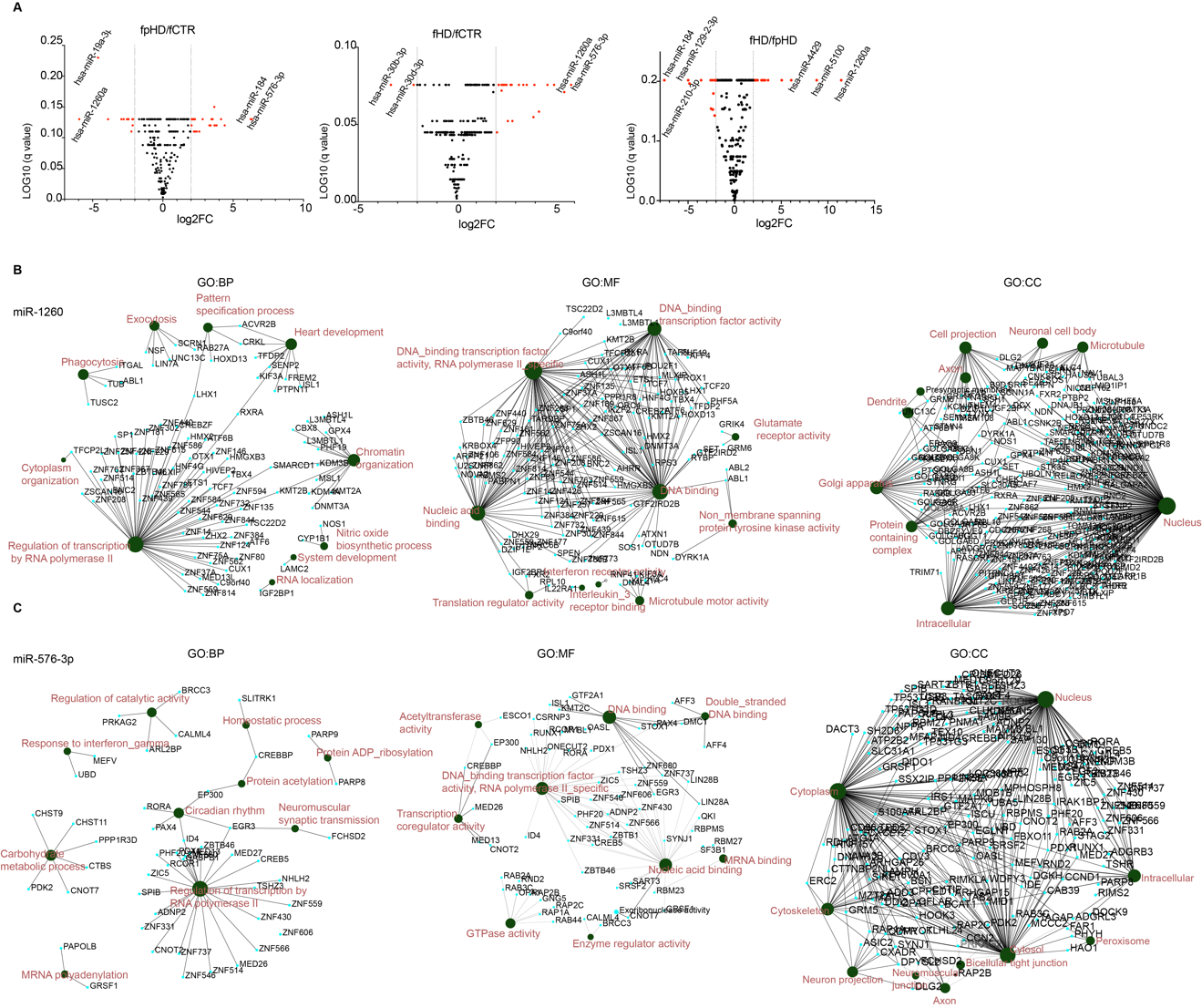


**Figure S3.** Differentially expressed miRNAs.

(A) Volcano plot of differently expressed miRNAs in EVs isolated from fCTR, fpHD and fHD cell lines, as assessed by microarray analysis. The horizontal axis represents the log2 ratio and the vertical axis represents log10 (q value). The red color indicates that the expression of the miRNA significantly increased/decreased by more than two-fold in EVs from the represented groups. (B-C) Enriched biological processes of miR-1260 family and miR-576-3p gene targets based on overrepresentation analysis (ORA). Gene set nodes are colored based on their enrichment p-value.

**Supplementary Table 1.** Metrics per sample along the different analysis steps.

| **Metrics /**  **samples** | **Total Reads**  **sequenced (#)** | **Total Reads after**  **QC (#)** | **Total Reads after**  **rRNA and tRNA**  **removal (#)** | **Reads mapped**  **to miRBase (#)** |
| --- | --- | --- | --- | --- |
| fCTR1 | 13,347,636 | 9,963,425 | 4,342,630 | 1,103,931 |
| fCTR2 | 13,152,667 | 5,324,116 | 2,492,835 | 687,854 |
| fCTR3 | 11,675,829 | 7,113,831 | 4,452,039 | 2,081,559 |
| fpHD1 | 13,392,005 | 8,171,010 | 5,260,904 | 404,498 |
| fpHD2 | 13,815,452 | 7,939,090 | 5,747,350 | 4,890,054 |
| fpHD3 | 11,245,268 | 7,058,606 | 4,499,043 | 238,631 |
| fHD1 | 11,121,267 | 4,307,082 | 1,821,026 | 1,077,743 |
| fHD2 | 9,725,087 | 4,574,519 | 3,095,192 | 2,576,082 |
| fHD3 | 10,327,642 | 6,174,631 | 3,632,983 | 2,856,454 |

Total Read Sequences – number of raw sequences obtained from the NextSeq sequencing platform; Total Reads after QC – number of reads after removing adapters and 4Ns introduced in library preparation; Total Reads after rRNA and tRNA removal against RFam – number of reads not corresponding to rRNA and tRNA; Reads mapped to MIRBase – number of reads mapped to MIRBase, version 22.

**Supplementary Table 2:** MiRNA differently expressed

**Supplementary Table 3:** miRDB target prediction data (related to figure 5)

**Supplementary Table 4:** Gene Ontology biological process enrichment analysis of differentially expressed genes (up/downregulated) for candidate genes identified through miRDB analysis

**Supplementary Table 5:** Intersection of the gene list from supplementary table 2 with SynGO annotated genes that uses list of all brain expressed genes as a default background set, defined as 'expressed in any GTEx v7 brain tissue'.

**Supplementary Table 6:** miRDB gene target prediction data for fCTR2 and fpHD3 and intersection of the gene list with SynGO annotated genes (related to figure 6)

**Supplementary Table 7:** Intersection of the gene list with SynGO annotated genes for fCTR2 and fpHD3 using the Gene Ontology Consortium database for two categories: GABAergic synapse (GO:0098982) and Synapse assembly (GO:0007416).
